# Colluvium supply in humid regions limits the frequency of storm-triggered landslides

**DOI:** 10.1038/srep34438

**Published:** 2016-09-30

**Authors:** Robert N. Parker, Tristram C. Hales, Simon M. Mudd, Stuart W. D. Grieve, José A. Constantine

**Affiliations:** 1 School of Earth and Ocean Sciences, Cardiff University, UK; 2 School of GeoSciences, University of Edinburgh, UK

## Abstract

Shallow landslides, triggered by extreme rainfall, are a significant hazard in mountainous landscapes. The hazard posed by shallow landslides depends on the availability and strength of colluvial material in landslide source areas and the frequency and intensity of extreme rainfall events. Here we investigate how the time taken to accumulate colluvium affects landslide triggering rate in the Southern Appalachian Mountains, USA and how this may affect future landslide hazards. We calculated the failure potential of 283 hollows by comparing colluvium depths to the minimum (critical) soil depth required for landslide initiation in each hollow. Our data show that most hollow soil depths are close to their critical depth, with 62% of hollows having soils that are too thin to fail. Our results, supported by numerical modeling, reveal that landslide frequency in many humid landscapes may be insensitive to projected changes in the frequency of intense rainfall events.

Where shallow landslides are present in soil-mantled landscapes they are the dominant mechanism of sediment transfer from hillslopes to channels^1^,^2^ and pose a significant hazard to life and property^3^,^4^. Most shallow landslides initiate during rainfall, leading to the suggestion that changes in the frequency and magnitude of rainfall events will have a significant, measureable effect on landslide frequencies^5^. Shallow landslides are translational landslides commonly formed in colluvium in unchanneled valleys (hollows)^6^ (Fig. 1). Rainfall and convergent subsurface water flow trigger shallow landslides by increasing pore pressures within the colluvium^7^. Most landslides remove colluvium down to the bedrock surface, after which the bare bedrock limits any further landsliding until colluvium thickens by transport from upslope^2^,^8^. Therefore, the landsliding rate is controlled by the frequency of rainfall events that produce pore pressures capable of initiating failure in each hollow and the rate of colluvium accumulation across the width of the hollow in the intervening time between rainfall events. The relative importance of these two controls on landslide triggering rate is poorly understood, in part due to the difficulty of determining the thickness of hollow material. Soil creep processes, such as tree throw or gopher burrowing, are thought to dominate sediment transport into hollows^2^. Measurements of rates of soil creep suggest that these processes are slow^9^ resulting in colluvium that accumulates in hollows over thousands of years^1^,^10^,^11^,^12^,^13^. Conversely, landslide-triggering storms in the Southern Appalachians recur at annual to decadal timescales^14^,^15^. Together this evidence suggests that accumulation is the major limitation on landslide frequency. Although at the regional scale there are always hollows becoming ready to fail somewhere in the landscape, individual hollows can only reproduce landslides on the timescale of thousands of years. The effect of slow colluvium accumulation is therefore to limit how frequently hollows become ready to fail across the landscape, and therefore limit the landslide frequency the landscape can sustain. If this observation is consistent across other soil-mantled mountains, it challenges the extent to which the number of landslides initiated during a particular storm is controlled by the magnitude of that storm^12^. Also, it suggests that landslide frequency may be insensitive to future changes in the frequency of extreme precipitation events. We examine the relationship between colluvium accumulation and pore pressure event frequency in 283 hollows in the Southern Appalachian Mountains. We assess the extent to which landslide frequency is limited by soil accumulation in this landscape. Using this analysis to constrain models of shallow landsliding in colluvial hollows, we test the sensitivity of landslide frequency to projected changes in future storminess.

For each hollow, we calculated the forces driving and resisting shallow landslide initiation and used these to calculate the pore pressure event size (expressed as the proportional saturation of the soil column) required to initiate a landslide. The shear component of the gravitational force, or weight of the colluvium, drives instability and depends on the local slope gradient and colluvium thickness. Resisting failure is the normal component of the gravitational force, modified by the pore pressure, the failure plane friction, usually approximated as the friction angle, and the additional cohesive strength that arises from a number of mechanisms including the electrostatic charges between platy minerals in the soil and plant roots^16^. Consistent with previous work, we account for the additive effect of lateral root cohesion as additional basal cohesion in the infinite slope model^17^, which is an appropriate solution for shallow landslides of this type^18^. For colluvium with cohesive strength, a minimum colluvium depth must be achieved before there is enough driving force to initiate a landslide. We call this the critical depth (*h*_*cr*_, Fig. S1). Where the soil depth is less than the critical depth, cohesive forces are such that if the colluvium fills with water, the pore pressure generated is not sufficient to cause a landslide^19^. Where intense precipitation creates pressure heads in excess of steady state pore pressures^7^, or where the exfiltration of additional water pressure from bedrock^20^ generates excess pore pressures, landslide triggering can occur at depths shallower than *h*_*cr*_. Given that the conditions required for this are highly variable in space, we have no reason to believe that the presence of excess pore pressures during rainstorms is ubiquitous across the landscape. Landslide scarp heights, measured at the sides of failed hollows do not fall below critical depths expected for pore pressures under saturated conditions (Fig. 2). If a significant amount of landslide triggering was driven by excess pore pressures through exfiltration or other processes, we would expect to see some of these landslide scarp heights at depths lower than their expected critical depth. That none of the landslide scarp heights fall below their critical depth supports the characterization of critical depth in terms of saturated colluvium in this and other studies^10^,^19^,^21^,^22^. Beyond the critical depth landslides can initiate where pore pressures are high enough to exceed the shear strength of the colluvium. This occurs due to a combination of increased antecedent moisture caused by convergent topographic flow and increased pressure heads due to locally intense rainfall^7^. Theoretically, colluvium could reach a maximum colluvium depth where it will fail under dry conditions due to its weight with additional strength provided by the negative (matric) water pressures^21^.

## Results

**Figure 2:**
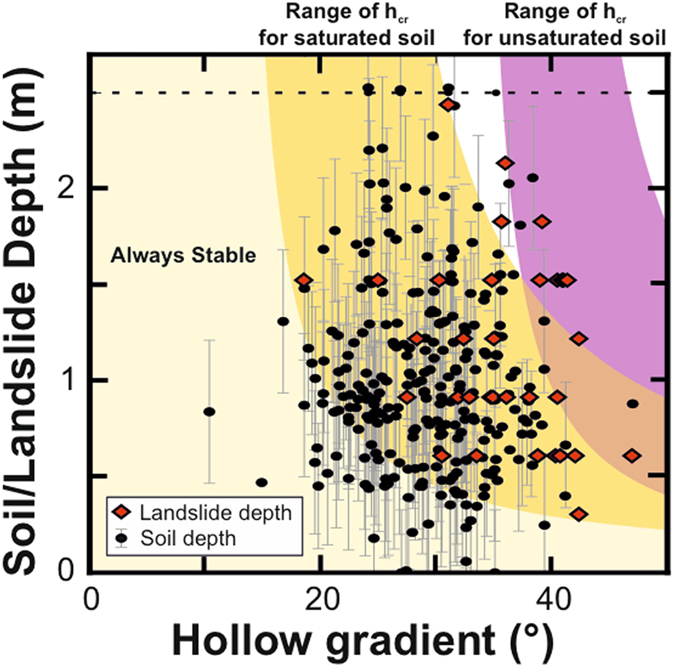
The distribution of Appalachian colluvium and landslide depths. Appalachian colluvial hollow depth data (black circles) as a function of hollow gradient, with error bars to show the standard deviation of uncertainty (Methods). Colluvial landslide depths (red diamonds) and hollow gradients attained from the North Carolina landslide database, measured in the field and accurate to the nearest 30 cm (1 foot)^4^. For each plot, we show the interquartile range of predicted critical colluvium depths based on random samples of slope, root and soil cohesion, soil friction angle, and saturated weights of soil. Most of our samples plot within the range of saturated *h*_*cr*_, suggesting that they are close to the critical colluvium depth, with some being located in high cohesion hollows that are stable, while others in low cohesion hollows are unstable. To determine the proportion of hollows that may be above or below their local critical colluvium depth we calculated the *h*_*cr*_ for the population of hollows by randomly assigning values for root and soil cohesion, soil friction angle, and saturated weights of soil. We then simulated the distribution of hollow depths relative to the critical colluvium depths 1000 times, to assess the potential error in this calculation.

**Figure 3:**
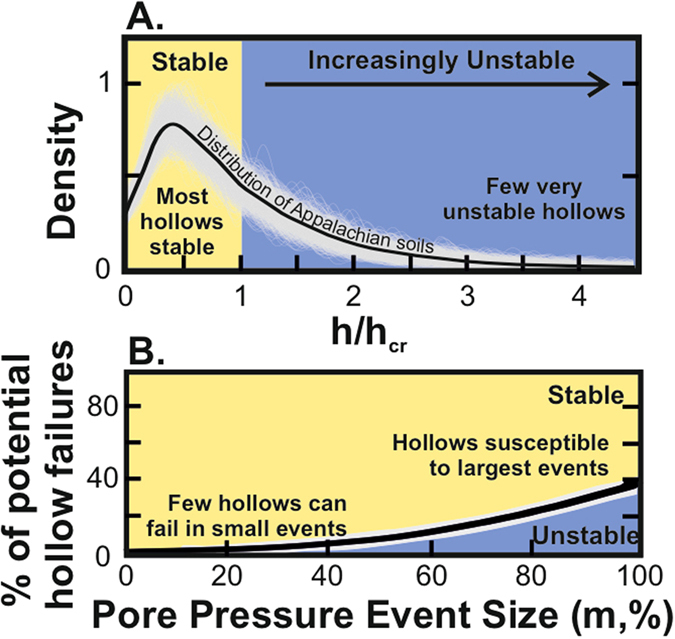
Calculations of Appalachian landslide potential. **(A**) The distribution of stable and unstable Appalachian slopes based on our Monte Carlo analysis. Stable slopes are calculated where colluvium depths (*h*) are lower than their critical colluvium depths (*h*_*cr*_). Each individual distribution of *h*/*h*_*cr*_ (gray lines) is calculated from randomly distributed soil strength parameters expressed as a kernel density (Kernel density was estimated using a Gaussian kernel, with the kernel bandwidth estimated using Scott’s Rule^45^). The black line averages the 1000 individual distributions. (**B**) Landslide potential expressed as the percentage of the total hollows that would fail in a colluvium saturation (pore pressure) event of particular size for the current Appalachian landscape. Very few hollows have attained colluvium depths that would cause them to fail in small pore pressure events, instead most landslides have to initiate in the largest pore pressure events.

Field-based measurements reveal that a large proportion of hollows in the Southern Appalachians have colluvium that is shallower than the critical colluvium depth. Colluvium depths (*h*) average 1.5 m for slopes between 20° and 40° (Fig. 2). We further constrained the distribution of hollow colluvium depths by calculating the range of critical colluvium depths for each hollow. Using a Monte Carlo simulation, we estimated the distribution of *h*/*h*_*cr*_ for randomly chosen parameters (Fig. 3A). Using this method, 62 ± 4% of hollows have colluvium depths below the critical value and cannot fail until the colluvium thickens further (Fig. 3A). We can further examine the data by investigating the pore pressure event size that would be required to initiate failure of the remaining 38 ± 4% of hollows in the landscape (Fig. 3B). This analysis suggests that only a very small proportion of hollows could possibly fail during pore pressure events that partially saturate the colluvium. For example, if a storm were to saturate half of the colluvium across all hollows in the landscape, it would trigger failure in no more than 7 ± 2% of them. Rainfall events with water tables in excess of 80% of the colluvium thickness are required to initiate landslides in ~20% of hollows, which accounts for around half of those in which colluvium is actually deep enough to fail. These results highlight that landslides in this landscape can only initiate during extremely large pore pressure events. By extension, rainfall events that produce a large number of landslides are only likely to occur when the water table in hollows exceeds 80% of colluvium thickness across a region.

The distribution of colluvium depths is consistent within a landscape where the landslide triggering rate is controlled by the accumulation rate of colluvium in hollows rather than the frequency of landslide-producing pore pressure events. A majority of hollows with colluvium depths below their critical depth suggest that the recurrence interval of storms is far shorter than the time taken for colluvium to accumulate to the critical depth. Comparing hollow accumulation rates and storm frequencies in the Appalachians lends further support to this argument. Hollow accumulation rates of between 0.05 and 0.7 mm/year (equivalent to 0.051 and 0.111 mm/year of bedrock lowering) were calculated from radiocarbon dating of two hollows within our field site^23^. At these rates, it takes between 1,500 and 20,000 years to accumulate 1 m of colluvium. In contrast, saturated conditions are common along hollow axes in the Southern Appalachians during large storms with relatively short return periods (10^0^ to 10^2^ years)^24^. While hollow soil moisture contents have not been measured directly in the Southern Appalachian Mountains, transient ecohydrological models provide some constraints on the frequency of hollow saturation events^24^. Soil moisture conditions for two catchments within our field area (Coweeta and Cartoogechaye) were modeled using the RHESSys model, constrained by stream flows and soil moisture records from each catchment. This modelling shows that during 2004, when landslide-producing Hurricanes Francis and Ivan occurred, 95% of hollow axes experienced full saturation^24^,^25^ (Fig. S6). The historical record of hurricanes and landsliding events provides secondary support for frequent high pore pressure events in hollows. For example, there have been 2 major landsliding events in the southern Appalachians that initiated hundreds to thousands of landslides; 1969 Hurricane Camille in Virginia and the 1940 unnamed hurricane in Deep Gap, North Carolina^3^.

Colluvium accumulation rates and depths have been measured in two other locations globally, the West Coast of the United States^1^,^26^,^27^,^28^,^29^,^30^ and the Shimane Prefecture in the south-west of the island of Honshu, Japan^10^,^11^,^12^. These sites and the Southern Appalachians span a range of mean annual precipitations (from 600–4000 mm/yr^10^,^25^,^29^,^30^) and long-term incision rates (0.03–0.9 mm/yr^31^,^32^) that encompass a wide range of landscapes where shallow landslides initiate. Hollow depths measured in the humid, forested Oregon and Washington Coast Ranges were <4 m in thickness and had radiocarbon charcoal ages of >1000 years^26^,^29^. In the Mediterranean climates of Northern California, these studies found some hollows contained deeper colluvium, consistent with less frequent storms^1^,^28^,^30^. Soil depths measured across ridges and hollows in Japan, show a similar pattern of shallow depths as in the Appalachians^10^. Modelling of hollow accumulation and stochastic storm frequency in Japan demonstrated that the timescale of colluvial accumulation was the primary limitation on landslide initiation^12^. Taken together these results support the argument that colluvium accumulation limits landslide frequency in humid, soil-mantled landscapes.

Shallow landslide frequency varies with time based on changes in three major controls: (i) the critical colluvium depth (*h*_*cr*_), which represents the minimum thickness by which a landslide can initiate. Critical colluvium depth is particularly sensitive to the magnitude of cohesion that can decrease during deforestation and other land use changes and potentially increase as weathering increases the proportion of clays. (ii) the depth of colluvium (*h*), which when combined with the critical colluvium depth provides an estimate of the magnitude of pore pressure event that will initiate a landslide; and (iii) rainfall, which drives the magnitude and frequency of pore pressure events^21^,^26^,^27^.

Using established solutions for incorporating colluvium accumulation into our landslide model^21^,^33^, we provide examples of how colluvium-supply limited landsliding is insensitive to projected future changes in precipitation. In the model, colluvium accumulates within a hollow assuming colluvial flux is linearly proportional to topographic gradient. The rate of colluvium accumulation depends on the difference in gradient between side slopes and the hollow axis and the transport coefficient, which reflects the efficiency of the mechanisms driving creep^33^. We estimate the transport coefficient (*D*) based on the measurements from humid soil-mantled landscapes^9^. Colluvium depth is reset to zero when the slope fails, consistent with observations that most landslides scour to bedrock in this landscape^4^,^6^.

First, we ran our model using 1000 synthetic hollows that had geometric and soil properties selected from the probability distributions of field-measured values (Fig. S4) using the Monte Carlo method. Taking the observation that colluvium depths in most hollows measured are close to their critical colluvium depth, we simulated landsliding for pore pressure events that filled the soil column at different frequencies. Here we utilise this simplified hydrology because storms that saturate the soil recur at shorter timescales than the colluvium accumulation timescales at which we are working (1000–10000 years). This allows us to understand the maximum possible effect that changes in storm frequency may have on a shallow landslide frequency, with respect to the limitation imposed by colluvium accumulation. Spatially distributed landslide assessments at a sub-annual timescale necessitate a more sophisticated characterization of hydrology^7^. However, at the long timescales of our experiment, the frequency of large precipitation events is the major hydrologic control on landslide frequency. We assume that root cohesions have not varied systematically through the Holocene because the Appalachians have been continuously forested, with a similar species composition^34^, suggesting a similar distribution of root cohesions to current forests^35^,^36^. Human activity over the past 200 years could possibly have affected root cohesions through deforestation and agricultural development. An assessment of the state of forest cover in 1900 showed that the Little Tennessee River Basin was 91% forested with only fertile alluvial plains cleared for agriculture, and no steep potential landslide sites deforested^37^. Much of the area was logged in 1919 for all stems larger than 15 inches at the stump, but has since been preserved by the U.S. Forest Service (our samples were all collected on USFS land)^38^. If there were significant historical landsliding in our sites, we would expect to see a preponderance of very shallow colluvium. We do not see this in our data, suggesting that while deforestation may have initiated some landslides, deforestation is unlikely to have initiated a regional-scale landsliding event. As there is no systematic difference in soil cohesions and friction angles between hollows with vastly different basal ages (20,000 years and 5,000 years^23^,^35^), we also assume that soil properties have not changed through time.

**Figure 4:**
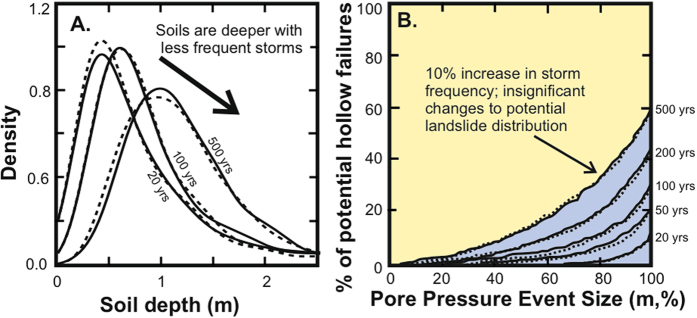
Simulated changes in landslide potential for different storm return periods and predicted changes in climate. (**A**) Modelled colluvium depth distributions for storms with different return periods (solid lines). Colluvium depths increase as return period increases and hollows have a longer time to infill. A 10% change in the return period of storms results in the dashed line. (**B**) The distribution of landslide potential for different modeled return periods (solid line) and 10% change in precipitation (dashed line). The result presented here uses the highest globally measured soil creep diffusivity for humid mountains (see Extended Data Fig. S5 for sensitivity analysis using the upper and lower bound diffusivity values).

To examine the relative importance of pore pressure event frequency and colluvium accumulation, we varied the frequency of pore pressure events that saturate the colluvium (between 20 and 500 years). We then calculated the colluvium depth distribution associated with that pore pressure event frequency. We simulated hollows with high colluvium accumulation rates by using a high value of diffusivity^9^. This analytical framework allows us to examine a system where climate has the largest possible influence on landslide frequency and colluvium depths (sensitivity analysis for different diffusivity values can be found in Fig. S5). In agreement with our field observations, our simulations produce colluvium depth distributions with average colluvium depths <1.5 m, with the average depth decreasing and the standard deviation increasing with less frequent storms (Fig. 4A). Mountains with long storm return periods should therefore contain deeper colluvium when compared to mountains that have frequent storms. In all of our simulations, the landslide potential remained low, with <60% of hollows in the landscape at depths above the critical colluvium depth (Fig. 4B).

Anthropogenic climate change projections based on general circulation models show that many humid landscapes on Earth will experience an increase of <10% in the frequency of extreme precipitation (defined as the maximum precipitation event with a return period of 20 years) by 2100^39^. Although the globally averaged frequency of tropical cyclones is projected to decrease by 6–34%, high resolution modelling studies project that the frequency of the most intense cyclones will increase. Increases in the global averaged intensity of tropical cyclones of 2–11% are also projected by 2100^40^. We used our model to simulate a 10% increase in storm frequency in line with globally averaged projections (Fig. 4).

This modeling exercise shows that the reduction in long-term landslide potential (percentage of sites with h > h_cr_) is insignificant and there is no measureable change in landslide frequency. The result does not change if we assume a more realistic hydrology (steady, slope parallel flow, based on D’Odorico and Fagherazzi^21^) and a probability distribution of rainfall events derived from modern rainfall records. We find that landslide frequency is even less sensitive to an increase in rainfall event frequency, than in our simplified model (Supplemental Figs S7 and S8). A 10% increase in rainfall event frequency results in at most a 0.1% reduction in landslide potential and a corresponding 0.3% increase in landslide frequency. At the upper limit of the projected shift to a wetter future climate, this 10% increase in frequency is combined with an 11% increase in precipitation intensity. In response to this change we see a 0.9% reduction in landslide potential and a corresponding 1.4% increase in landslide frequency. This insensitivity of the landscape to increasing precipitation frequency and intensity is because the return period of landslide-triggering storms is higher than time required for colluvium to accumulate above the critical depth. Using reasonable assumptions about storm return periods and the potential changes to this with anthropogenic climate change, we argue that the frequency of shallow landslides in soil-mantled mountains may be insensitive to changes in precipitation extremes. Our observations are consistent with those made in humid mountains in Japan and the Western United States, suggesting that colluvium-supply limited landsliding may be a ubiquitous characteristic of soil-mantled mountain landscapes.

## Methods

We measured the distribution of colluvium depths across a portion of the Little Tennessee River Basin, part of the soil-mantled Southern Appalachian Mountains, USA We measured 257 hollow colluvium depths from excavation pits and soil tile probe measurements (described in the supplementary information) randomly sampled from all hollows across an area of 1340 km^2^ (Fig. 2). We measured colluvium depths using different methods (Fig. S3). Exact hollow colluvium depths were derived from soil pits dug to the colluvium-saprolite boundary. Taking the maximum of 20 soil tile probe measurements, we were able to attain depths with a standard deviation error of 0.33 m. Similarly, taking the maximum of 3 soil tile probe measurements, we attained depths with a standard deviation error of 0.37 m. These data were supplemented with 52 measurements of the thickness of shallow landslide escarpments measured by the North Carolina Geological Survey^4^. These provide a colluvium depth for the edges of shallow landslides, and represent a minimum colluvium depth prior to failure.

We calculated the critical colluvium depth at each of our sites using the infinite slope method commonly applied to translational slides. This method assumes that during failure a uniform thickness of colluvium is removed along a slope of constant angle and infinite extent^16^. The infinite slope assumption is generally considered valid for natural landslides, where the landslide length is long relative to the depth^18^. Using this model the critical colluvium depth depends on the slope angle, measured at the surface using a 6m airborne Light Detection and Ranging (LiDAR) elevation model^41^ and the soil strength parameters including friction angle, soil and root cohesion and the saturated weight of soil. Friction angles, soil cohesions and saturated weights were measured in the field and laboratory^35^. We measured lateral root cohesions in 27 soil pits within naturally forested plots^35^,^36^. These soil properties have been measured within individual hollows in the Appalachian landscape. Rather than sample the soil properties of other hollows from an assumed distribution (commonly a uniform distribution is assumed), we randomly sampled from the measured parameter distributions shown in Fig. S4 using the Monte Carlo method. Hence for each hollow we established the range of possible critical colluvium depths based on the range of soil strength parameters observed in Appalachian forests.

Note that due to the sensitivity of landslide initiation to hollow colluvium depth and the critical colluvium depth, accurate characterization of these variables is essential for meaningful predictions of future landsliding. Given the difficulty of measuring colluvium depth, landslide models typically assume a constant value or a uniform distribution for this and other material parameters^42^. Our data show that this may be a valid assumption for friction angle, saturated weight, and soil cohesion, but not for the right-skewed root cohesion and colluvium depth distributions (Fig. S4). This difference between our measured distributions and typical model assumptions occurs in the two parameters for which the limit equilibrium model is extremely sensitive and highlights the necessity of accurate characterization of these parameters in hazard modeling.

To further support our findings, we also include results generated using a fully-implemented steady-state hydrologic model, across a subset catchment (Coweeta Long-term Ecological Research Laboratory) using a sample of 6068 hollows delineated from 1m LiDAR data, using the DrEICH algorithm^43^. This model accounts for the additional complexity of drainage area and colluvium depth-dependent hydrologic response to rainstorms, and rainfall consistent with the short modern record. To demonstrate the implications of colluvium-supply limited landsliding in the context of future climate change, we ran our model to simulate global, upper-bound projected increases in precipitation. For realistic predictions of future landslide activity, our results emphasize the importance of combining consensus precipitation projections from downscaled climate models^44^, with shallow landslide models incorporating colluvium accumulation and precipitation triggering.

## Additional Information

**How to cite this article**: Parker, R. N. *et al*. Colluvium supply in humid regions limits the frequency of storm-triggered landslides. *Sci. Rep.* **6**, 34438; doi: 10.1038/srep34438 (2016).

## Supplementary Material

Supplementary Information

## Figures and Tables

**Figure 1:**
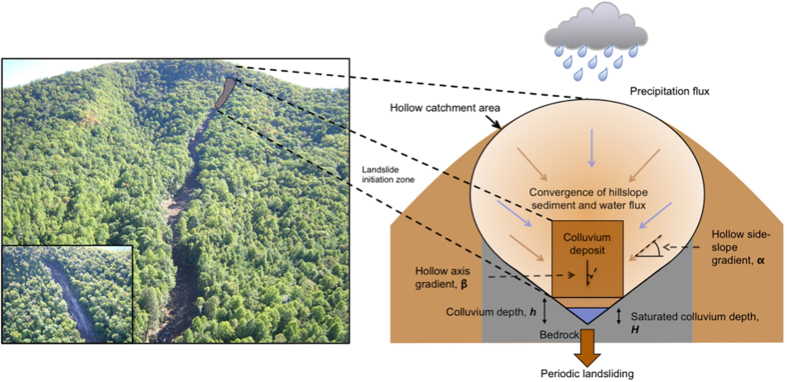
Shallow colluvium landslides in North Carolina. (Left) Photographs of the 2004 Peeks Creek landslide, triggered by intense precipitation during Hurricane Ivan (photos courtesy of Rick Wooten). (Right) Schematic diagram of colluvial hollow landslide recharge and triggering model.
