# Supplementary material for: Colluvium supply in humid regions limits the frequency of storm-triggered landslides
Source: Sci Rep. 2016 Sep 30;6:34438. doi: 10.1038/srep34438 (PMC5043370; doi:10.1038/srep34438)
Supplement: Supplementary Information [file srep34438-s1.pdf]

Title: Colluvium supply in humid regions limits the frequency of storm-triggered landslides

Authors: Robert N. Parker[1]*, Tristram C. Hales[1], Simon M. Mudd[2], Stuart W. D. Grieve[2], José A. Constantine[1]

Supporting Information

Study area

Our field site is located in Macon County (1,347 km^2), North Carolina, U.S.A., part of the Southern Appalachian Mountains. The whole range is soil-mantled, with upland hillslopes characterized by the nose and hollow topography typical of Appalachian regions[1]. The geology of Macon County is composed of high and moderate grade metamorphic rocks in a structurally complex arrangement that crosses topography[2]. The mountain range is tectonically quiescent, with some debate as to the timing of late stage uplift of the mountains[3]. Regardless of their genesis, the mountains maintain high relief of close to 1000 m through high topographic features such as the Blue Ridge and Nantahala Escarpments, resulting in the steep topography necessary to generate significant landsliding. The soil mantle is maintained by a humid, sub-tropical climate at lower elevations and marine, humid, temperate climate at higher elevations, with mean annual precipitation ranging between 1800 and 2300 mm for elevations between 700 and 1400m[4].

Current forests in the landslide prone higher elevations of the Southern Appalachians are dominated by either northern hardwoods, or a combination of xeric oak-pine, cove and mixed hardwood forests[5]. The current forest structure is thought to have been established at close to its current elevation distribution by the mid-Holocene[6]. Prior to this, more extensive northern hardwood forests existed, and during the last glacial maximum the highest peaks are likely to have been dominantly periglacial[7]. Empirical observations of root reinforcement of soils have shown that there is a difference in the strength of soils between noses and hollows[8]. Root reinforcement within individual hollows is highly variable [8-10] due primarily to differences in root biomass of different tree species, and within individual tree species as a function of age, substrate, nutrient contents and other factors. There is no regional pattern in root reinforcement

provided by the dominant forest types in the Appalachians[8]. However, there may be significant uncertainty in the root strength of an individual hollow, which we constrain within our model. Because there is no obvious regional trend in root reinforcement across the forest types of the Southern Appalachians, we infer that forest change alone does not cause significant differences in root reinforcement through time. The later 19th and early 20th century saw extensive deforestation in this area both by clearfelling and selective logging[11]. However, the only study of deforestation effects showed no difference in landslide initiation rates between clearfelled and natural forests, suggesting that clearfelling did not significantly increase the proportion of the landscape susceptible to landsliding[12].

Landslides have been recorded in the southern and central Appalachians for over a century[13]. Hundreds of landslides have been associated with large cyclonic storms in North Carolina, Virginia and West Virginia between 1916 and 2007. Tens of landslides across Macon County were associated with 2004 Hurricanes Ivan and Frances. The resulting investigation by the North Carolina Geological Survey (NCGS) led to a 2-year-long historical, remote sensing, and field study that created an extensive landslide inventory for the area[14-16]. Field measurements of recently failed landslides (2003-2013) were used as part of our dataset of soil information.

Soil depth measurements

We calculated the distribution of current hollow colluvium thicknesses in the field using a combination of soil pits and soil tile probe measurements. We randomly chose hollows to survey by examining areas of convergent topography with potential in the categories Lower bound instability, Upper bound instability, Unconditionally unstable, from SINMAP analysis undertaken by the NCGS[15].

In order to measure the depth of colluvium in large numbers of hollows, we developed a field methodology using a 2.5 meter long, AMS soil tile probe (http://www.benmeadows.com/ams-heavy-duty-extendible-tile-probe_36814889/). This is a reinforced steel rod that can be driven by hand into rugged soils, to attain a bedrock refusal depth. Depths were measured vertically and rotated normal to the hillslope surface using the local slope gradient. The technique provides an accurate estimate of colluvium depth in soft soil with a discrete bedrock interface. However, underestimates occur where the probe strikes hard clasts in the soil column, and overestimates

occur where the probe penetrates into bedrock fractures or zones of rock that have weathered to saprolite. We developed a methodology with three levels of accuracy for measuring soil depth.

At each site we first probed the soil around the apex of the hollow, to find the area of deepest colluvium. Excavating a pit in the hollow apex, down to the bedrock, attained the most accurate and definitive measure of soil depth. The soil thickness from the soil surface to the bedrock interface was then measured using a tape measure. Accounting for uneven soil and bedrock surfaces, we estimate the accuracy of this technique to be $\sigma = \pm 0.02$ m.

Using the soil tile probe, our most accurate measure of soil depth was attained from the maximum of 20 probe depths, collected in a 1x1 m sample zone in the apex of the hollow ($h_{probe_{20}\max}$). Monte Carlo analysis using data from our pilot study indicated that this technique should provide 95% confidence that the depth measurement was within 10% of the actual soil depth. We assessed the final accuracy and precision of this method through comparison of these data with the depth of colluvium measured definitively in excavation pits (at 16 sites), using regression analysis (Extended Data Fig. 3). On average our $h_{probe_{20}\max}$ data overestimate the colluvium depth by 5%, and we attain a standard deviation of residuals of 0.33 m:

(1)

$$h = 0.95 h_{probe_{20}\max} \pm \sigma = 0.33$$

Our coarsest, reconnaissance level measure of soil depth was attained by taking the maximum of 3 probe depths, within a 1x1 m sample zone in the hollow apex ($h_{probe_3\max}$). We assess the uncertainty for these sites through Monte Carlo simulation of our methodology, inverse-transform sampling the maximum of 3 depths from data at the same 16 pit sites. Our results suggest that the $h_{probe_3\max}$ data underestimate the colluvium depth by 15%, and for these sites we attain a standard deviation of residuals of 0.37 m.

(2)

$$h = 1.17 h_{probe_3\max} \pm \sigma = 0.37$$

Using (1) and (2), we transformed our probe depth data, to estimate the depth and depth uncertainty for each hollow.

Critical Soil Depth Measurements

Critical soil depths (h_{cr}) were calculated using the Mohr-Coulomb failure criterion solved for depth and assuming full soil saturation,

(3)

$$h_{cr} = \frac{c}{\gamma_w \tan\phi \cos\beta + \gamma_{sat} \cos\beta (\tan\beta - \tan\phi)} .$$

where c is the soil and root cohesion, γ_w is the weight of water, γ_{sat} is the saturated weight of soil, ϕ is the friction angle, and β is the slope of the hollow[17]. We determined the key parameters from field and laboratory observations and using a digital elevation model:

Root cohesions (c) were determined by analyzing the diameter distribution and tensile strength of roots collected in pits excavated in Coweeta Hydrologic Laboratory[8,18]. Using the Wu method[19] we determined the lateral cohesion at each pit.

Soil cohesion and friction angles (ϕ) were measured for two soil pits in Coweeta Hydrologic Laboratory[8]. Samples were triaxially tested by the North Carolina Department of Transport and parameters were calculated based on the stress path methodology.

Saturated weight of soil (γ_{sat}) was measured during the emplacement of time-domain reflectivity probes following the methods of Amoozegar[20].

Hollow axis gradients (β) were constrained using a 6 m resolution LiDAR-derived digital elevation model [21]. Landscape gradients were derived at the DEM resolution by calculating the maximum gradient between each 6 m pixel and its 8 neighbouring pixels. We attained β from the DEM gradient at the GPS location (accurate to <6 m) of each sample site.

The rate of soil accumulation in hollows is determined by the ratio of *hollow axis gradient* to the *hollow side-slope gradient* and the soil creep transport coefficient (D) (see (5) below). *Hollow side-slope gradients* (α) were attained by taking the hypotenuse (Euclidean maximum) of the hollow axis gradient *(β),* and the slope gradient measured perpendicular to the hollow axis. Note that for hollows, by definition, α is always greater than β, so we report this variable in terms of a β/α hollow concavity ratio.

Soil creep transport coefficient (D) values for the Southern Appalachians have been estimated at 6.5-10 m^2 ka^{-1} [22], based on in-situ and meteoric ^{10}Be analysis of hillslope soils [23].

To assess the uncertainty in critical soil depth measurements we used the Monte Carlo Method. We randomly sampled the distributions of input variables (Extended Data Fig. 4 A-E) using inverse transform sampling. This technique interpolates between quantiles of our sampled data, allowing us to generate continuous random variables without being restricted to the sample values.

One-dimensional Model of Hollow Infilling and Evacuation

We modelled infilling and evacuation for a synthetic population of 1000 hollows (Extended Data Fig. 2 & 3) with characteristics derived randomly from our field and DEM parameters (Extended Data Fig. 4 A-E). We model infilling and evacuation in colluvial hollows in one-dimension using a model developed by Dietrich et al.[24] and D'Odorico and Fagherazzi [25]. This model simplifies hollow geometry and hydrology by assuming that there is little change in slope along the hollow axis, therefore soil accumulation is determined by the difference in side-slope and hollow gradients. This model is preferred over more complicated models of hollow infilling and evacuation because the result is a measure of soil depth that can be directly compared to our data. The model estimates the soil depth for a population of hollows based on two components: i) the growth of colluvial deposits via weathering of underlying bedrock and hillslope sediment transport processes, ii) downslope evacuation of colluvium during landslides, promoted by pore-pressure generation during rainstorms.

The growth of colluvial deposits in hollows is modelled assuming soil creep is linearly proportional to the topographic gradient. Given a hollow where sediment enters from side slopes and leaves along the hollow axis[23], this results in:

(4)

$$\frac{dh}{dt} = \frac{K}{2h},$$

and

(5)

$$K = 2D\cos\beta(\tan^2\alpha - \tan^2\beta),$$

where h is the colluvium depth (measured perpendicular to the bedrock), t is time, β is the hollow axis gradient and α is the hollow side-slope gradient measured along the soil-bedrock interface, and D is the sediment transport coefficient [26]. Assuming that the underlying hollow bedrock geometry does not vary substantially with time, β and α remain constant [25]. The cross sectional shape of each by each hollow is assumed to be triangular and colluvium thickness increases as

(6)

$$h = \sqrt{Kt}.$$

Soil production by bedrock weathering beneath the hollow is assumed to be negligible with respect to infilling via soil diffusion from the hollow side-slopes [24]. When hollows fail, landslide events scour the colluvium down to bedrock, such that $h = 0$. This assumption is supported by observational evidence that shallow landslide failure surfaces generally coincide with the regolith-bedrock interface [27-29].

The stability of colluvium accumulated in hollows is modelled using the Mohr-Coulomb failure criterion applied to an infinite planar slope [30]. This one-dimensional technique is widely used as a geotechnical component in geomorphic and landscape evolution models. The infinite slope assumption is generally considered valid for natural landslides, where the landslide length is long relative to the depth [31]. Uncertainty analyses suggest that where length-depth ratios exceed 25, stability (factor of safety) predictions from more physically accurate finite-element models converge within 5% of those from the infinite slope method [32]. This criterion is therefore applicable to shallow landslides in colluvial hollows, and provides an appropriate level of accuracy for assessing hollow behavior at the regional-scale. Additionally, more accurate models are not justified, due to the lack of knowledge on the soil geotechnical and hydrological properties and their spatial variability [33]. The form of the infinite-slope model used and the implications for hollow behavior discussed below, are specific to soils with cohesion. The apparent cohesion provided by roots is also necessary to explain the presence of slopes greater than maximum values of ϕ observed in Appalachian soils. We take the approach of many other

authors (e.g. Schmidt et al., 2001[34]) and calculate the additional cohesion provided by roots as the lateral cohesion provided by root penetrating the soil column. Thus, the use of this model is valid for the study of shallow landslides in our field area.

For each hollow we calculate the critical failure depths for partially saturated soils (h_{crp}) for a particular percentage soil saturation; the height of the water-table as a fraction of the soil thickness above the bedrock interface (m).

(7)

$$h_{crp} = \frac{c}{m\gamma_w \tan\phi \cos\beta + \gamma_{sat} \cos\beta(\tan\beta - \tan\phi)}$$

Here it is assumed that the subsurface flow is uniform with hydraulic gradient corresponding to the topographic slope. The hydrologic model that we infer is the standard model that forms the basis of most shallow landslide models [25,35]. Measurements of high exfiltration pressures in a shallow landslide in Coos Bay, Oregon suggest groundwater pressures may affect this condition[36], however, no shallow landslide model parameterizes the bedrock exfiltration pressure component of pore pressure. Assuming that saturated overland flow takes place when the height of the water column exceeds the soil depth, the saturated depth cannot be greater than the colluvium thickness, such that when $h < h_{cr}$, the colluvium is always stable. The maximum soil depth (h_{max}) is the depth of colluvium at which a hollow will become unstable regardless of pore pressure state. However, h_{max} is only relevant to the behavior of hollows with $\beta > \phi$, where an increase in colluvium depth favors instability of the slope. Where $\beta \leq \phi$, an increase in colluvium thickness favors slope stability and h_{max} $h_{max}$is infinite. In other words, the saturated soil depth required to trigger failure increases as the soil thickens.

Influence of changes in storm frequency on landlside frequency using a steady-state hydrologic model

To further support our findings, we also include results generated using a fully-implemented steady-state hydrologic model, across a subset catchment (Coweeta Long-term Ecological Research Laboratory) using a sample of 6068 hollows delineated from 1m LiDAR topographic data, using the DrEICH algorithm[37]. After D'Odorico and Fagherazzi (2003)[38], the precipitation into a hollow is equated to the outgoing subsurface flow occurring through the saturated depth:

(8)

$$RA = {H_{sat}}^{2} K_{sat} \sin\beta \frac{1}{\tan\delta}$$

where R is the rainfall intensity, K_{sat} is the hydraulic conductivity ($K_{sat} \sin\beta$ is the specific discharge of the subsurface flow (Darcy's law in the assumption of uniform flow), A is the hollow catchment area, and $\tan\delta$ represents the ratio of saturated height to width at the triangular outlet of the hollow (δ is the slope gradient at 90° to the hollow axis). The saturated depth can be then expressed as:

(9)

$$H_{sat} \sqrt{\frac{RA}{K_{sat} \sin\beta \frac{1}{\tan\delta}}}$$

K_{sat} was set to 65 md^{-1} (after [39]), which results in a long-term distribution of modelled landslide potential consistent with that observed in hollows where we measured colluvium depth. Although K_{sat} exhibits a high level of natural variability, and ranges over several orders of magnitude for soils of different textures, this value provides a calibration of landslide potential consistent with our observations, and therefore appropriate for testing the sensitivity of landslide potential and frequency to increases in precipitation.

To test the sensitivity of landslide potential and frequency to a 10% increase in precipitation event frequency, we first generated synthetic annual maximum precipitation events from a locally observed 75-year daily precipitation record (Fig. 7A) and elevation-dependent conversion ratios[40]. The distribution of daily precipitation intensities is expressed as a gamma function fitted to the observed data, which we find to best characterise the observed data out of all available continuous distributions (http://docs.scipy.org/doc/scipy-0.16.0/reference/stats.html, Fig. 7B). The synthetic timeseries of largest annual storms was then generated by taking the maximum of 365 randomly selected daily precipitation intensities for each year. This distribution corresponds closely with the observed distribution of annual maximum daily intensities between 1937 and

2012, suggesting that this technique provides a reasonable representation of long-term precipitation patterns in this landscape (Fig. 7C).

Using the same parameters as in our simplified simulations, we then ran the model for a spin up period of 300,000 years to allow landslide frequency and landslide potential variables to stabilize. For a further 40,000 years, we first continued the simulation with no change in precipitation frequency (Fig. 8A). Then, using the same precipitation event series, we reran the simulation for the last 40,000 years, but decreased the model time step by 10%, to simulate a 10% increase in precipitation frequency (Fig. 8B). Comparing the results, we find that a 10% increase in precipitation event frequency results in a 0.1% reduction in landslide potential and a corresponding 0.3% increase in landslide frequency. At the upper limit of the projected shift to a wetter future climate, this 10% increase in frequency is combined with an 11% increase in precipitation intensity. In response to this change we see a 0.9% reduction in landslide potential and a corresponding 1.4% increase in landslide frequency (Fig. 8C). Despite the increase in long-term landslide frequency, we also find that the maximum or peak numbers of landslides triggered by individual storms are reduced, as more frequent, larger storms increasingly limit the accumulation of surplus landslide potential in the landscape.

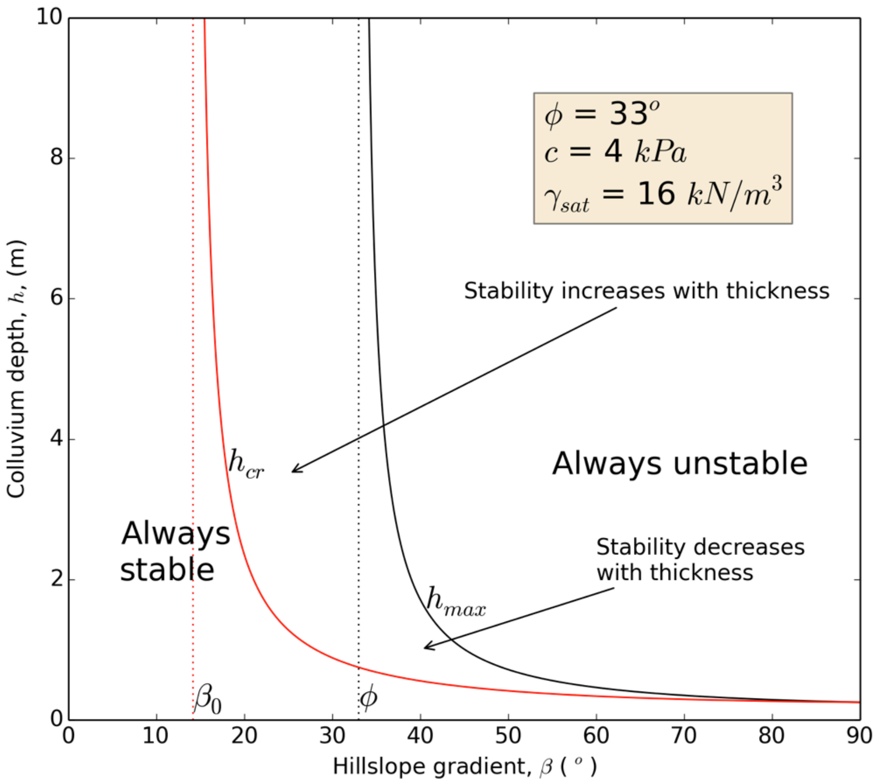

Fig. 1. Slope stability as a function of slope gradient and colluvium depth, for some typical Appalachian soil strength parameters.

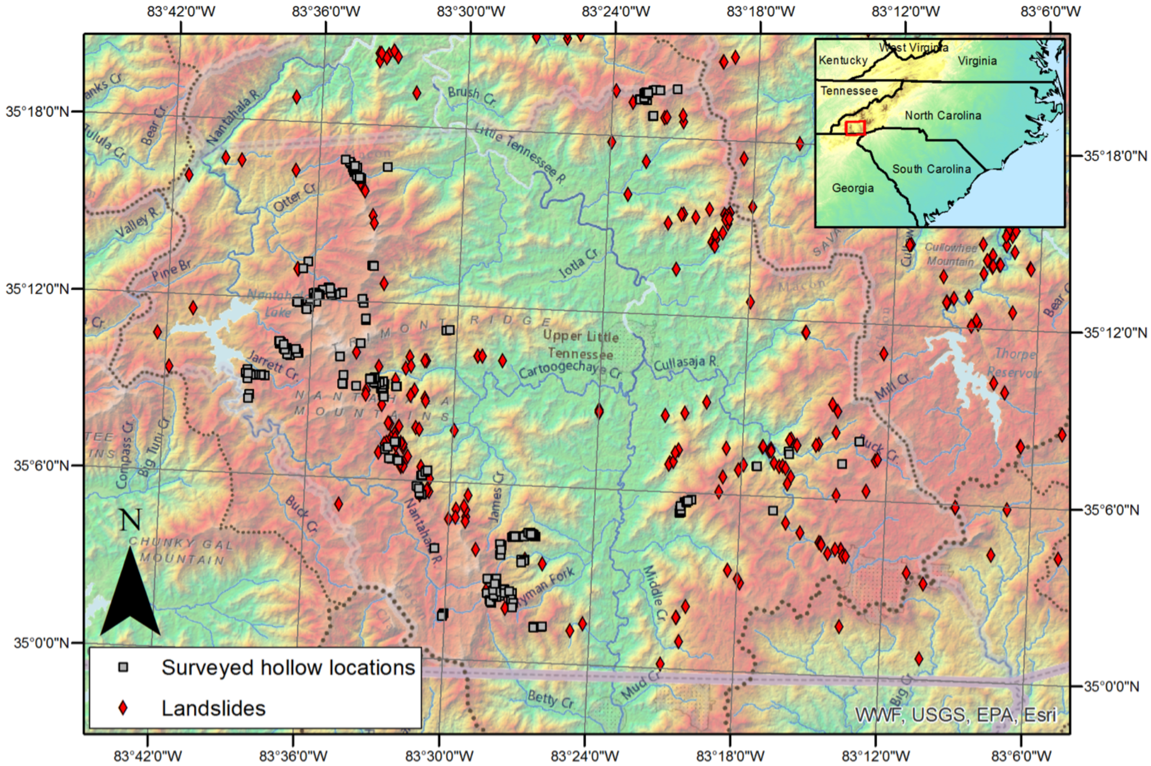

Fig. 2. Field area relief map of Macon County, North Carolina (USA), showing locations of 257 surveyed hollows and 52 shallow landslides from the North Carolina landslide database [41]. Map was generated using ArcMap 10.2.1 (http://desktop.arcgis.com/en/arcmap).

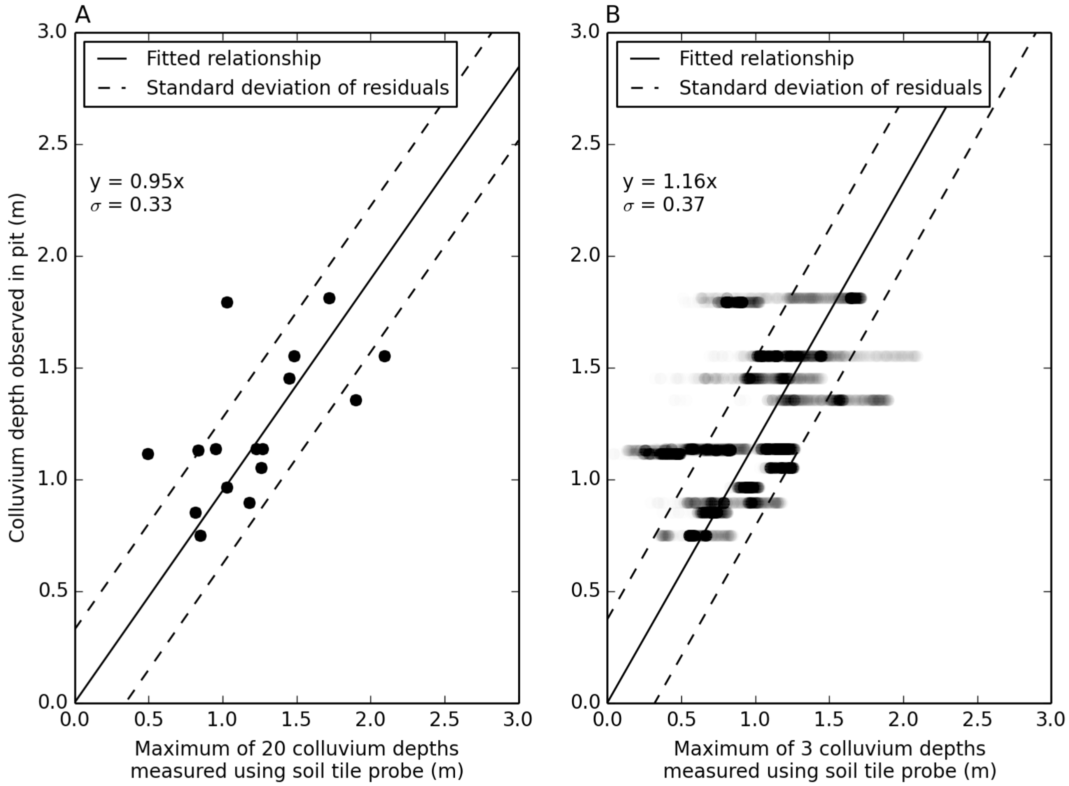

Fig. 3 Uncertainty of soil-tile-probe-estimated colluvium depths, as a function of definitive colluvium depths measured in excavation pits. A) Data attained from the maximum of 20 probed depths, B) Data attained from the maximum of 3 probed depths (generated via Monte Carlo simulation using data shown in A).

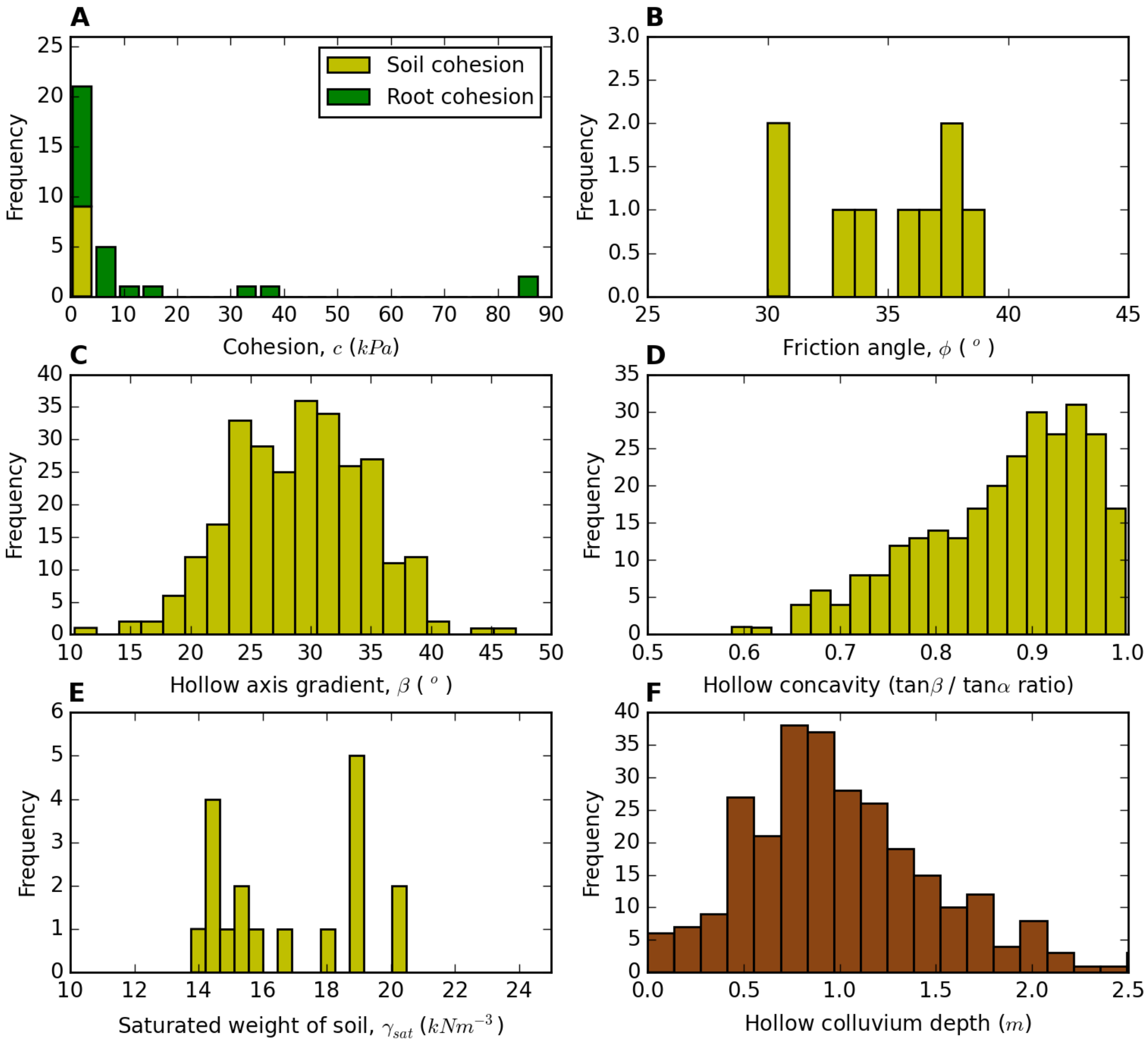

Fig. 4. Model input distributions of hillslope material properties, hollow geometry and collvuial depths constrained for Appalachian colluvial hollows. Note that the use of a hollow concavity variable allows the gradient of hollow side-slopes to be expressed as a function of the hollow axis gradient, where the hollow concavity is the ratio of the hollow axis gradient to the hollow side-slope gradient. In this way these two components of the hollow geometry – axis and side-slope gradients - are varied co-dependently rather than independently, producing distributions of hollow geometries consistent with our observed hollows.

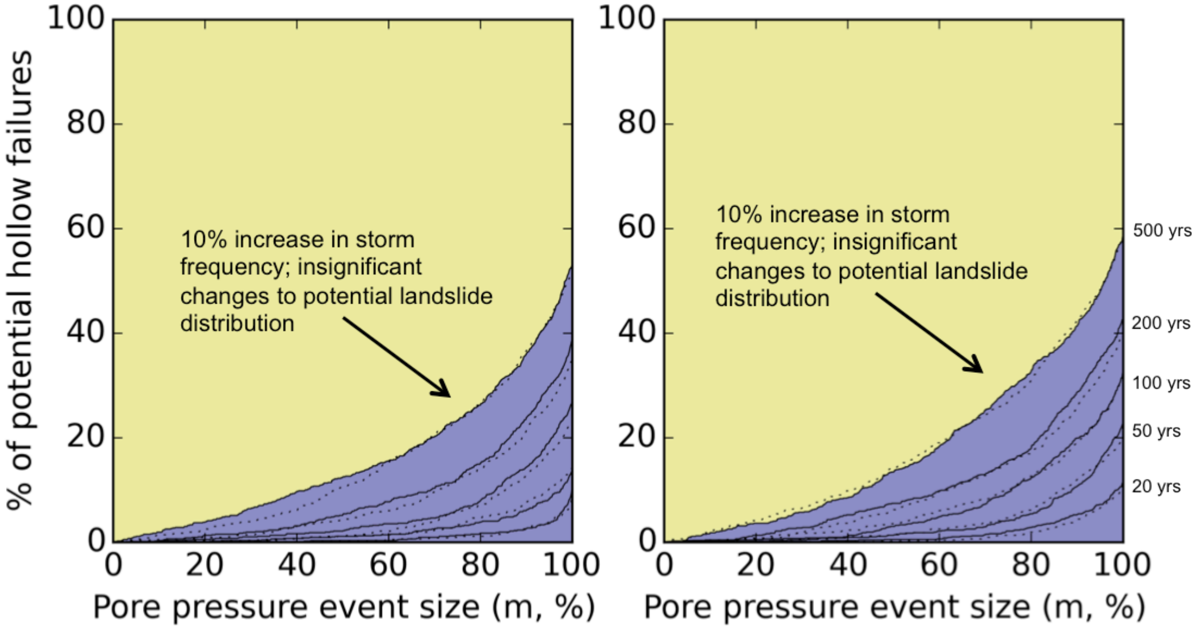

Fig. 5. Plots of landslide potential as a function of pore pressure event size, for upper and lower bound estimates of soil creep transport coefficient (D) for the Southern Appalachian Mountains[23]. Using the same return periods as shown in Fig. 3. (A) $D = 6.5$. (B) $D = 10.0$.

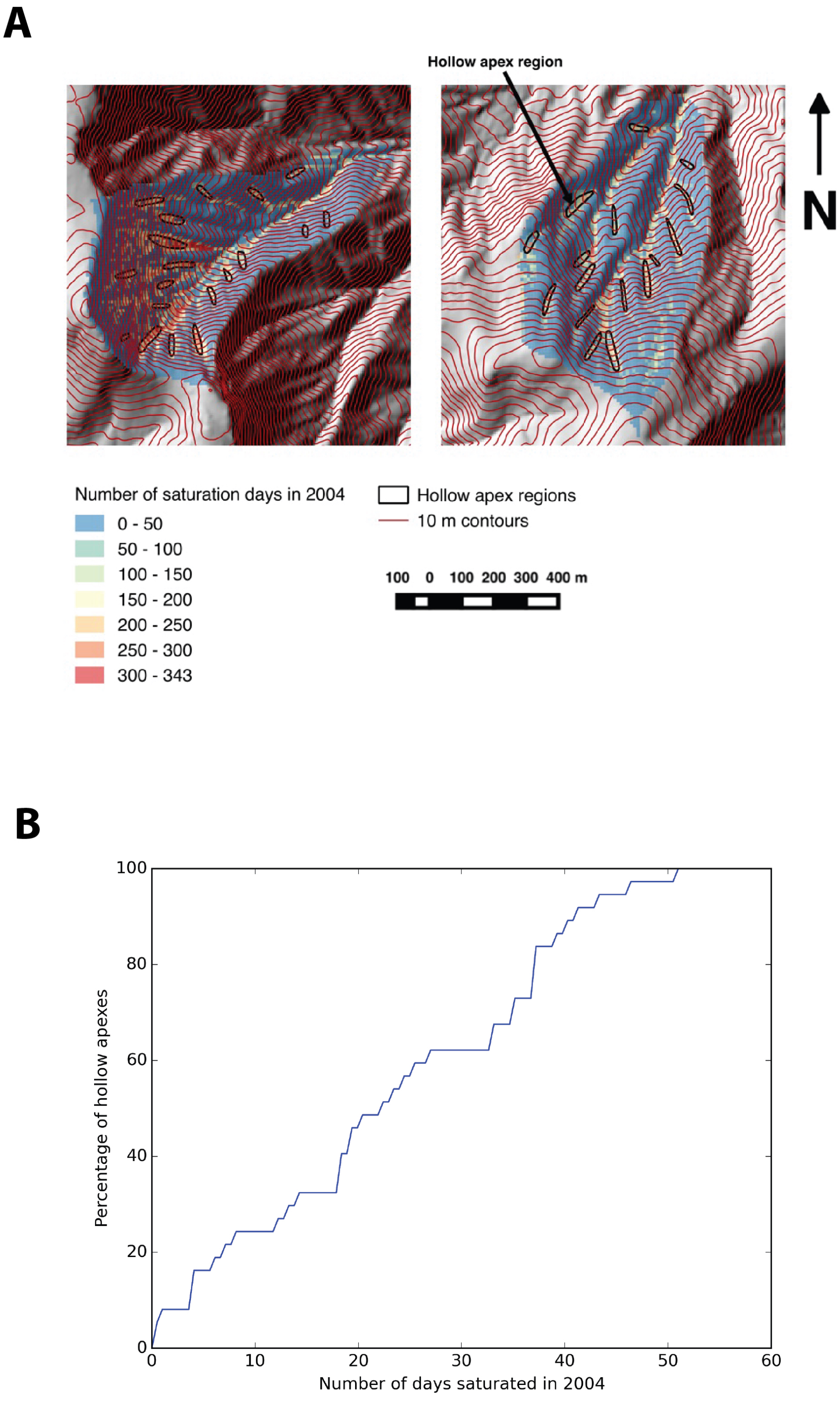
A
Hollow apex region
N
Number of saturation days in 2004
0 - 50
50 - 100
100 - 150
150 - 200
200 - 250
250 - 300
300 - 343
Hollow apex regions
10 m contours
100 0 100 200 300 400 m
B
Percentage of hollow apexes
Number of days saturated in 2004
0
20
40
60
80
100
0
10
20
30
40
50
60

Fig. 6: A: Maps of the number of soil saturation days for two Southern Appalachian catchments in our study area, derived from ecohydrological modelling using RHESSys for 2004, when landslide-producing Hurricanes Francis and Ivan occurred [40]. Hollow apex regions have been mapped through interpretation of 1 and 6 m LiDAR topographic data. Maps were generated using QGIS 2.12.0-Lyon (https://www.qgis.org). B: Cumulative distribution of the number of days on which hollow apex regions are completely saturated. During 2004, 95 % of hollow axes display full saturation.

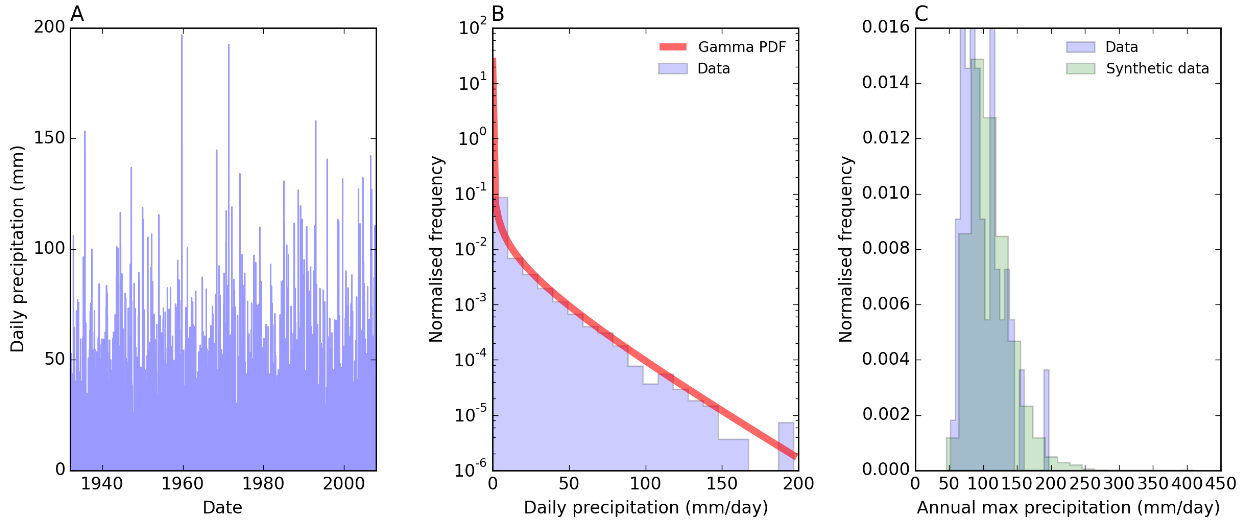

Fig. 7: Precipitation data used in model simulations. A) Daily precipitation record taken from the Coweeta LTER from 1937 to 2012. (http://climhy.lternet.edu/plot.pl) B) Distribution of daily precipitation from A, showing a fitted gamma distribution. C) Synthetic annual maximum daily precipitation distribution, generated from the gamma distribution shown in B, with observed maximum annual daily precipitations shown for comparison.

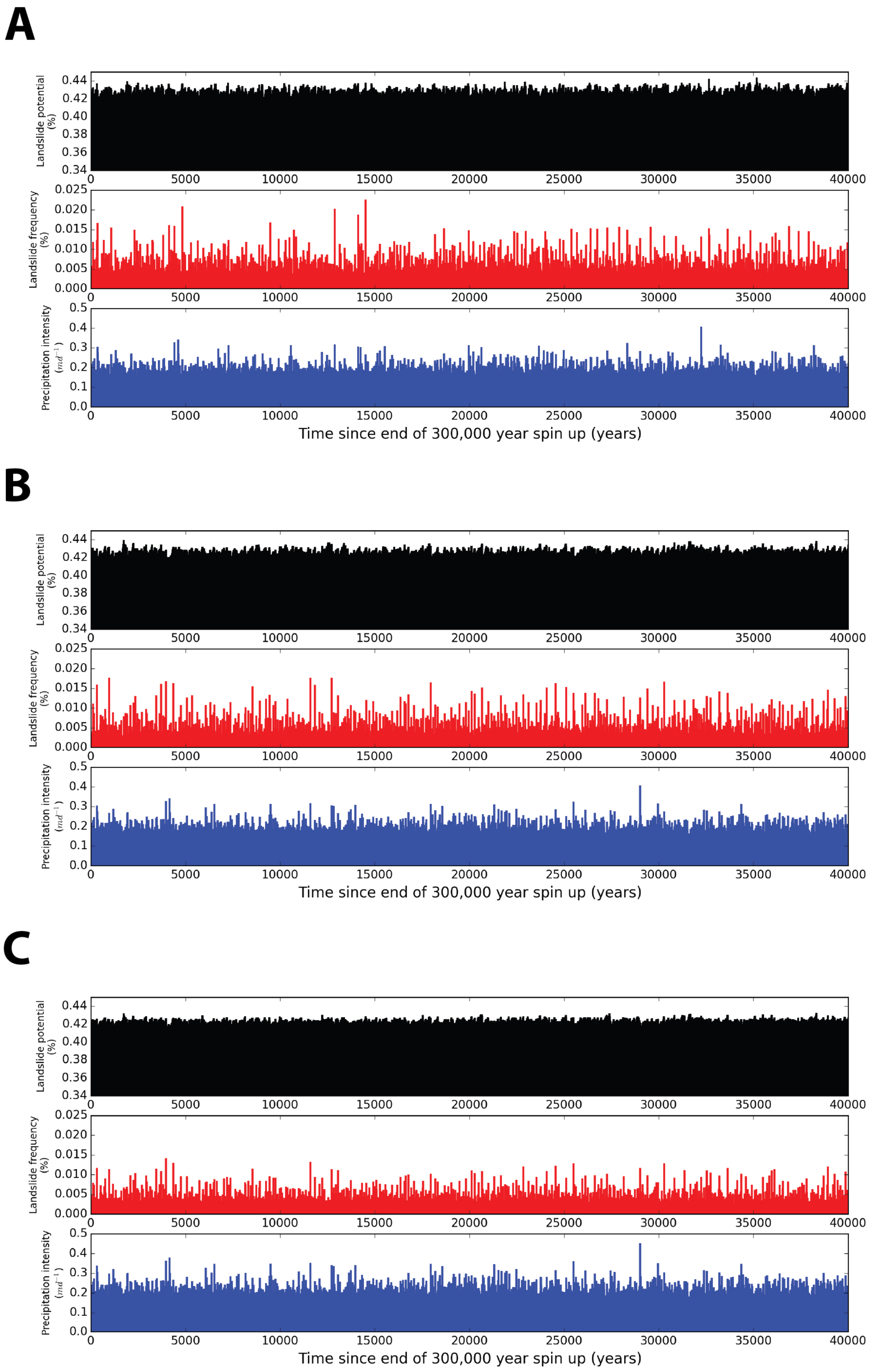
A
B
C
Landslide potential (%)
0.44
0.42
0.40
0.38
0.36
0.34
Landslide frequency (%)
0.025
0.020
0.015
0.010
0.005
0.000
Precipitation intensity (md⁻¹)
0.5
0.4
0.3
0.2
0.1
0.0
0
5000
10000
15000
20000
25000
30000
35000
40000
Time since end of 300,000 year spin up (years)

Fig. 8: A: Timeseries of synthetic precipitation events, landslide frequency and landslide potential. B: Same timeseries as in A, with a 10 % decrease in model time-step, to simulate a 10 % increase in precipitation event frequency. C: Same timeseries as in A, with a 10 % decrease in model time-step to simulate a 10 % increase in precipitation event frequency, and an 11 % increase in model precipitation intensity.

References and Notes:

1 Hack, J. T. & Goodlett, J. C. Geomorphology and forest ecology of a mountain region in the central Appalachians. *USGS Professional Paper* **347**, 66 (1960).

2 Hatcher, R. D. The Coweeta Group and Coweeta syncline: Major features of the North Carolina-Georgia Blue Ridge. *Southeastern Geology* **21**, 17-29 (1979).

3 Gallen, S. F., Wegmann, K. W. & Bohnenstiehl, D. R. Miocene rejuvenation of topographic relief in the southern Appalachians. *GSA Today* **23**, doi: 10.1130/GSATG1163A.1131 (2013).

4 Swift, L. W., Cunningham, G. B. & Douglass, J. E. in *Forest Hydrology and Ecology* (eds W.T. Swank & D.A. Crossley) 35-55 (Springer, 1988).

5 Bolstad, P. V., Swank, W. T. & Vose, J. M. Predicting Southern Appalachian overstory vegetation with digital terrain data. *Landscape Ecology* **13**, 271-283 (1998).

6 Delcourt, H. R. Late Quaternary vegetation history of the eastern Highland Rim and adjacent Cumberland Plateau of Tennessee. *Ecological Monographs* **49**, 255-280 (1979).

7 Nelson, K. J. P., Nelson, F. E. & Walegur, M. T. Periglacial Appalachia: paleoclimatic significance of blockfield elevation gradients, eastern USA. *Permafrost and Periglacial Processes* **18**, 61-73 (2007).

8 Hales, T. C., Ford, C. R., Hwang, T., Vose, J. M. & Band, L. E. Topographic and ecologic controls on root reinforcement. *Journal of Geophysical Research* **114**, doi:10.1029/2008JF001168 (2009).

9 Roering, J. J., Schmidt, K. M., Stock, J. D., Dietrich, W. E. & Montgomery, D. R. Shallow landsliding, root reinforcement, and the spatial distribution of trees in the Oregon Coast Range. *Canadian Geotechnical Journal* **40**, 237-253 (2003).

10 Schwarz, M., Cohen, D. & Or, D. Spatial characterization of root reinforcement at stand scale: Thoery and case study. *Geomorphology* **171-172**, 190-200 (2012).

11 Douglass, J. E. & Hoover, M. D. in *Forest Hydrology and Ecology at Coweeta* (eds W.T. Swank & D.A. Crossley) 17-31 (Springer, 1988).

12 Eschner, A. R. & Patric, J. H. Debris avalanches in Eastern upland forests. *Journal of Forestry* **80**, 343-347 (1982).

13 Clark, G. M. Debris slide and debris flow historical events in the Appalachians south of the glacial border. *Reviews in Engineering Geology* **7**, 125-138 (1987).

14 Wooten, R. M. *et al.* Geologic, geomorphic, and meteorological aspects of debris flows triggered by Hurricanes Frances and Ivan during September 2004 in the Southern Appalachian Mountains of Macon County, North Carolina (southeastern USA). *Landslides* **5**, 31-44 (2008).

15 Wooten, R. M. *et al.* in *N.C. Geological Survey Geologic Hazards Map Series 1* (Raleigh, North Carolina, 2006).
16 Latham, R. S., Wooten, R. M. & Reid, J. C. in *Proceedings of the 56th Highway Geology Symposium.* 277-290.
17 Selby, M. J. *Hillslope materials and processes*. 2 edn, (Oxford University Press, 1993).
18 Hales, T. C. & Miniat, C. F. Hillslope-scale root cohesion driven by soil moisture conditions. *Earth Surface Processes and Landforms*, in review (2016).
19 Wu, T. H., McKinnell III, W. P. & Swanston, D. N. Strength of tree roots and landslides on Prince of Wales Island, Alaska. *Canadian Geotechnical Journal* **16**, 19-34 (1979).
20 Amoozegar, A. A compact constant-head permeameter for measuring saturated hydraulic conductivity of the vadose zone. *Soil Science Society of America Journal* **53**, 1356–1361 (1989).
21 North Carolina Flood Mapping Program. 6 m LiDAR elevation model. (2014).
22 Hurst, M. D., Mudd, S. M., Yoo, K., Attal, M. & Walcott, R. Influence of lithology on hillslope morphology and response to tectonic forcing in the northern Sierra Nevada of California. *Journal of Geophysical Research: Earth Surface* **118**, 832-851 (2013).
23 Jungers, M. C. *et al.* Tracing hillslope sediment production and transport with in situ and meteoric 10Be. *Journal of Geophysical Research: Earth Surface* **114**, F04020, doi:10.1029/2008JF001086 (2009).
24 Dietrich, W. E., Wilson, C. J. & Reneau, S. L. in *Hillslope Processes* (ed A. D. Abrahams) 361–388 (Allen and Unwin, 1986).
25 D'Odorico, P. & Fagherazzi, S. A probabilistic model of rainfall-triggered shallow landslides in hollows: A long-term analysis. *Water Resources Research* **39** (2003).
26 Culling, W. E. H. Analytical Theory of Erosion. *Journal of Geology* **68**, 336-344 (1960).
27 O'Loughin, C. L. & Pearce, A. J. Influence of Cenozoic geology on mass movement and sediment yield response to forest removal, North Westland, New Zealand. *Bull. Int. Assoc. Eng. Geol.* **14**, 41-46 (1976).
28 Sidle, R. C. & Swanson, D. N. Analysis of a small debris slide in coastal Alaska. *Can. Geotech. J.* **19**, 167-174 (1982).
29 Trustrum, N. A. & De Rose, R. C. Soil depth-age relationship of landslides on deforested hillslopes, Taranaki, New Zealand. *Geomorphology* **1**, 143-160 (1988).
30 Terzaghi, K. & Peck, R. B. *Soil mechanics in engineering practice*. (Wiley Intersci, 1967).
31 Haneberg, W. C. A rational probabilistic method for spatially dis- tributed landslide hazard assessment. *Environmental and Engineering Geoscience* **10** (2004).
32 Milledge, D. G., Griffiths, D. V., Lane, S. N. & Warburton, J. Limits on the validity of infinite length assumptions for modelling shallow landslides. *Earth Surface Processes and Landforms* **37**, 1158-1166, doi:10.1002/esp.3235 (2012).
33 Sidle, R. C., Pearce, A. J. & O'Loughlin, C. L. *Hillslope Stability and Land Use*. (AGU, 1985).
34 Schmidt, K. *et al.* The variability of root cohesion as an influence on shallow landslide susceptibility in the Oregon Coast Range. *Canadian Geotechnical Journal* **38**, 995-1024 (2001).
35 Tarolli, P. & Tarboton, D. G. A new method for determination of most likely landslide initiation points and the evaluation of digital terrain model scale in terrain stability mapping. *Hydrology and Earth System Sciences* **10**, 663-677 (2006).

36 Montgomery, D. R., Schmidt, K. M., Dietrich, W. E. & McKean, J. Instrumental record of debris flow initiation during natural rainfall: Implications for modeling slope stability. *Journal of Geophysical Research: Earth Surface (2003–2012)* **114** (2009).

37 Clubb, F. J., Mudd, S. M., Milodowski, D. T., Hurst, M. D. & Slater, L. J. Objective extraction of channel heads from high-resolution topographic data. *Water Resources Research* **50**, 4283-4304, doi:10.1002/2013WR015167 (2014).

38 D'Odorico, P. & Fagherazzi, S. A probabilistic model of rainfall-triggered shallow landslides in hollows: A long-term analysis. *Water Resources Research* **39**, 1262, doi:10.1029/2002WR001595 (2003).

39 Montgomery, D. R., Sullivan, K. & Greenberg, H. M. Regional test of a model for shallow landsliding. *Hydrological Processes* **12**, 943-955, doi:10.1002/(SICI)1099-1085(199805)12:6<943::AID-HYP664>3.0.CO;2-Z (1998).

40 Hwang, T. *et al.* Simulating vegetation controls on hurricane-induced shallow landslides with a distributed ecohydrological model. *Journal of Geophysical Research: Biogeosciences*, 2014JG002824, doi:10.1002/2014JG002824 (2015).

41 Wooten, R. M. *et al.* in *Geological hazards map series 1* (2006).
